# Comparison of the Outcomes of Individuals With Medically Attended Influenza A and B Virus Infections Enrolled in 2 International Cohort Studies Over a 6-Year Period: 2009–2015

**DOI:** 10.1093/ofid/ofx212

**Published:** 2017-10-07

**Authors:** Dominic E Dwyer, Ruth Lynfield, Marcelo H Losso, Richard T Davey, Alessandro Cozzi-Lepri, Deborah Wentworth, Timothy M Uyeki, Fred Gordin, Brian Angus, Tavs Qvist, Sean Emery, Jens Lundgren, James D Neaton, INSIGHT Influenza Study Group, Bitten Aagaard, Álvaro H D Borges, Alessandro Cozzi-Lepri, Marius Eid, Per O Jansson, Marianne Jeppesen, Zillah Maria Joensen, Ruth Kjærgaard Pedersen, Jens Lundgren, Birgit Riis Nielsen, Mary Pearson, Lars Peters, Tavs Qvist, Brian Angus, Abdel Babiker, Rachel Bennett, Nafisah Braimah, Yolanda Collaco-Moraes, Adam Cursley, Fleur Hudson, Sarah Pett, Charlotte Russell, Helen Webb, Dianne Carey, David Courtney-Rodgers, Sean Emery, Pamela Shaw, Fred Gordin, Adriana Sanchez, Barbara Standridge, Michael Vjecha, Kate Brekke, Megan Campbell, Eileen Denning, Alain DuChene, Nicole Engen, Michelle George, Merrie Harrison, James D Neaton, Ray Nelson, Siu-Fun Quan, Terri Schultz, Deborah Wentworth, John Baxter, Shawn Brown, Marie Hoover, John Beigel, Richard T Davey, Robin Dewar, Erin Gover, Rose McConnell, Julia Metcalf, Ven Natarajan, Tauseef Rehman, Jocelyn Voell, Dominic E Dwyer, Jen Kok, Timothy M Uyeki, David Munroe, Damian Aguila, Maria Fernanda Alzogaray, Maria Fernanda Ballesteros, Laura Barcan, Laura Barcelona, Waldo Belloso, Veronica Berdiñas, Pablo Bonvehi, Juan Pablo Caeiro, Veronica Cisneros, Ana Crinejo, Daniel David, Luz Doldan, Juan Ebenrstejin, Flavio Lipari, Ana Lopardo, Gustavo Lopardo, Marcelo Losso, Pablo Lucchetti, Sergio Lupo, Laura Moreno Macias, Alejandra Moricz de Tesco, Analia Mykietiuk, Estaban Nannini, Gabriel Nieto, Laura Nieto, Luciana Peroni, Ignacio Retta, Patricia Rodriguez, Marisa Sanchez, Pablo Sanchez, Mariana de Paz Sierra, Silvina Tavella, Elena Temporiti, Liliana Trape, Ines Vieni, Eduardo Warley, Diego Yahni, Abel Humberto Zarate, Anchalee Avihingsanon, Kanlaya Charoentonpuban, Ploenchan Chetchotisakd, Peeraporn Kaewon, Naphassanant Laopraynak, Weerawat Manosuthi, Kanitta Pussadee, Opass Putcharoen, Kiat Ruxrungtham, Gompol Suwanpimonkul, Sasiwimol Ubolyam, Roberto Arduino, Barbara Atkinson, Taryn M Aulicino, Jason V Baker, Cindy Bardascino, Caitlin Bass, John D Baxter, Mark Beilke, Beverly D Bentley, Mary Lee Bertrand, Ann B Brown, June Carbonneau, Richard Cindrich, Patty Coburn, Calvin J Cohen, Linda Clark, Shirley Cummins, Paul Dassow, Jack A DeHovitz, Nila J Dharan, Leslie Faber, Marti Farrough, Matthew Freiberg, Edward Gardner, Kimberly Jo Garrett, Christiane Geisler, Marshall Glesby, Julia Green, Joanne Grenade, Edie Gunderson, John Gunter, Kirsis Ham, Susan Holman, Valery Hughes, Christopher Hurt, Mary Johnson, Glory Koerbel, Susan Koletar, Audrey Lan, Rodger MacArthur, Cheryl Marcus, Norm Markowitz, Maria Laura Martinez, Karen McLaughlin, Raquel Nahra, Mary Jane Nettles, Daniel Nixon, Richard Novak, Kathleen Nuffer, Hannah B Olivet, Bola Omotosho, Armando P Paez, Marta Paez-Quinde, Sonija Parker, Namrata Patil, Hari Polenakovik, Sandra Powell, Rachel A Prosser, Nancy A Reilly, Paul F Riska, Stacey Rizza, Robert Schooley, Marla Schwarber, James Scott, Gary L Simon, Jon Sivoravong, Daniel J Skiest, Clemencia Solorzano, Rita Sondengam, Nicole Swanson, Ellen Tedaldi, Zelalem Temesgen, Doug Thomas, Bill Thron, Colleen Traverse, David E Uddin, Daniel Z Uslan, Marina Vasco, William M Vaughan, Isabel Vecino, Barbara Wade, Catrice Walker, Kathy Watson, Vicky Watson, David Wohl, Cameron R Wolfe, Leslie Andry, Mireille Bielen, Nathan Clumeck, Eric Florence, Kabamba Kabeya, Jolanthe Sagaer, Jozef Weckx, Olga Anagnostou, Anastasia Antoniadou, George Daikos, Vicky Gioukari, Ioannis Kalomenidis, Maria Kantzanou, Georgios Koratzanis, Nikolaos Koulouris, Efstratios Maltezos, Symeon Metallidis, Vlassis Polixronopoulos, Helen Sambatakou, Athanasios Skoutelis, Giota Touloumi, Nikolaos Vasilopoulos, Mark Bloch, Nicky Cunningham, Dominic E Dwyer, Sian Edwards, Julian Elliott, Jill Garlick, Philip Habel, Fiona Kilkenny, Helen Lau, Karen MacRae, John McBride, Richard Moore, Isabel Prone, Ristila Ram, Sue Richmond, Norm Roth, Tuck Meng Soo, Thompson Jo-Anne, Trina Vincent, Emanuel Vlakahis, Rachel Woolstencroft, David Chadwick, Tristan Clarke, Jane Democratis, David Dockrell, Robert Heyderman, Ben Jeffs, Stefan Kutter, Martin Llewelyn, Jane Minton, Melanie Newport, Ashley Price, Carlos Benites, Raul Castillo, Romina Chinchay, Eva Cornelio, Maria Guevara, Luis Gutierrez, Jose Hidalgo, Alberto La Rosa, Yvett Pinedo, Maria Saenz, Juan Vega, Bente Baadegaard, Karen Bach, Philippa Collins, Jan Gerstoft, Lene Hergens, Lene Pors Jensen, Zillah Maria Joensen, Gitte Kronborg, Iben Rose Loftheim, Henrik Nielsen, Lars Oestergaard, Court Pedersen, Jens Aage Stauning Pedersen, Yordanos Yehdego, Frank Bergmann, Christoph Boesecke, Johannes R Bogner, Norbert Brockmeyer, Christine Czaja-Harder, Rika Draenert, Gerd Fätkenheuer, Hartwig Klinker, Tim Kümmerle, Clara Lehmann, Vera Müller, Andreas Plettenberg, Jürgen Rockstroh, Stefan Schlabe, Wolfgang E Schmidt, Dirk Schürmann, Gundolf Schüttfort, Ulrich Seybold, Christoph Stephan, Albrecht Stoehr, Klaus Tillmann, Susanne Wiebecke, Timo Wolf, Jose Arribas, Javier Carbone, Eduardo Fernández Cruz, David Dalmau, Vincente Estrada, Patricia Herrero, Hernando Knobel, Paco López, Rocío Montejano, José Sans Moreno, José Ramón Paño, Begoña Portas, Maria Rodrigo, Pilar Romero, Domingo Sánchez-Sendín, Vincente Soriano, Elzbieta Bakowska, Andrzej Jerzy Horban, Brygida Knysz, Karolina Pyziak Kowalska, Anna Zubkiewicz-Zarebska, Kerstin Kase, Helen Mülle, Kai Zilmer, Gladys Allendes, Jimena Flores, Rebeka Northland, Carlos Perez, Isabel Velasco, Marcelo Wolff, Man-Yee Chu, Tak-chiu Wu, Heinz Burgmann, Selma Tobudic, Mayumi Imahashi, Junji Imamura, Yasumasa Iwatani, Ayumi Kogure, Masashi Nakahata, Wataru Sugiura, Yoshiyuki Yokomaku, Anne Maagaard

**Affiliations:** 1 Institute of Clinical Pathology and Medical Research, NSW Health Pathology, Westmead Hospital, Westmead, Sydney, Australia; 2 Minnesota Department of Health, St. Paul, Minnesota; 3 Hospital J.M. Ramos Mejía, Buenos Aires, Argentina; 4 National Institute of Allergy and Infectious Diseases, National Institutes of Health, Bethesda, Washington, DC; 5 University College London, London, UK; 6 Division of Biostatistics, School of Public Health, University of Minnesota, Minneapolis, Minnesota; 7 Influenza Division, US Centers for Disease Control and Prevention, Atlanta, Georgia; 8 Veterans Affairs Medical Center, Washington, DC; 9 Nuffield Department of Medicine, University of Oxford, Oxford, UK; 10 Department of Infectious Diseases, Rigshospitalet, Copenhagen, Denmark; 11 Kirby Institute, University of New South Wales, Sydney, Australia; 12 Department of Infectious Diseases, Rigshospitalet, Copenhagen, Denmark; 13 Division of Biostatistics, School of Public Health, University of Minnesota, Minneapolis, Minnesota

**Keywords:** follow-up, influenza A(H1N1)pdm09, influenza A(H3N2), influenza B, outcomes

## Abstract

**Background:**

Outcome data from prospective follow-up studies comparing infections with different influenza virus types/subtypes are limited.

**Methods:**

Demographic, clinical characteristics and follow-up outcomes for adults with laboratory-confirmed influenza A(H1N1)pdm09, A(H3N2), or B virus infections were compared in 2 prospective cohorts enrolled globally from 2009 through 2015. Logistic regression was used to compare outcomes among influenza virus type/subtypes.

**Results:**

Of 3952 outpatients, 1290 (32.6%) had A(H1N1)pdm09 virus infection, 1857 (47.0%) had A(H3N2), and 805 (20.4%) had influenza B. Of 1398 inpatients, 641 (45.8%) had A(H1N1)pdm09, 532 (38.1%) had A(H3N2), and 225 (16.1%) had influenza B. Outpatients with A(H1N1)pdm09 were younger with fewer comorbidities and were more likely to be hospitalized during the 14-day follow-up (3.3%) than influenza B (2.2%) or A(H3N2) (0.7%; *P* < .0001). Hospitalized patients with A(H1N1)pdm09 (20.3%) were more likely to be enrolled from intensive care units (ICUs) than those with A(H3N2) (11.3%) or B (9.8%; *P* < .0001). However, 60-day follow-up of discharged inpatients showed no difference in disease progression (*P* = .32) or all-cause mortality (*P* = .30) among influenza types/subtypes. These findings were consistent after covariate adjustment, in sensitivity analyses, and for subgroups defined by age, enrollment location, and comorbidities.

**Conclusions:**

Outpatients infected with influenza A(H1N1)pdm09 or influenza B were more likely to be hospitalized than those with A(H3N2). Hospitalized patients infected with A(H1N1)pdm09 were younger and more likely to have severe disease at study entry (measured by ICU enrollment), but did not have worse 60-day outcomes.

Influenza A(H1N1)pdm09 virus emerged in 2009 [1], and it now co-circulates with influenza B and A(H3N2) viruses. Comparisons of different influenza types/influenza A subtype infections are useful to understand clinical outcome variations, including intensive care unit (ICU) admission, mortality, and severity predictors. Ideally, such data would come from follow-up studies of geographically diverse populations enrolled over multiple successive influenza seasons. In addition to assisting clinical management and preparedness responses, such data could inform clinical trial design for new therapeutics.

Several studies describe the clinical characteristics, hospital admission rates, and severity of hospitalized cases for patients with laboratory-confirmed influenza by virus type/subtype [2–6]. US population–based hospitalization surveillance reported that adults with influenza A(H1N1)pdm09 were more likely to require ICU admission than those with A(H3N2) or B, although mortality did not vary [2, 3]. Neither ICU admission nor mortality differed by virus type/subtype among French inpatients [4]. In 30-day follow-up of outpatients in Wisconsin, hospitalization for both adults and children with influenza A vs B [5] and with A(H1N1)pdm09 vs A(H3N2) [6] were similar. These studies were limited by sample size; the outpatient studies only enrolled from 1 region.

To compare demographics, clinical characteristics and follow-up outcomes for adults infected with influenza A(H1N1)pdm09, A(H3N2), or B viruses, data from 2 prospective, concurrent, multicenter inpatient and outpatient cohorts were analyzed. The studies enrolled in northern and southern hemisphere sites from 2009 through 2015, with laboratory confirmation of influenza virus type/subtype, thus allowing comparison of infections over multiple seasons and locations.

## MATERIALS AND METHODS

In 2009, the International Network for Strategic Initiatives in Global HIV Trials (INSIGHT) initiated 2 international observational cohort studies for adults aged ≥18 years with influenza A(H1N1)pdm09 virus infection: FLU002 for outpatients with influenza-like illness (ILI) and FLU003 for individuals hospitalized with complications and enrolled from either the general ward or the ICU [7, 8]. Protocols were amended to include other influenza viruses (June 2011), and in October 2013 FLU003 was amended to remove the eligibility requirement of having severe or complicated influenza at enrollment. Some sites chose to enroll only outpatients or inpatients. Institutional review boards approved the protocols, and individual signed informed consent was obtained.

Enrollment nasal/oropharyngeal swabs were collected and sent to central laboratories (Leidos, MD; ABML, NJ) for influenza A(H1N1)pdm09, A(H3N2), and B virus testing by reverse transcriptase polymerase chain reaction (RT-PCR). Only RT-PCR-confirmed influenza infections were analyzed [8].

FLU002 outpatients had 14-day follow-up for death and hospitalization. FLU003 inpatients had a 60-day follow-up, with disease progression outcomes defined differently for general ward (death, hospitalization ≥28 days, ICU admission, or use of mechanical ventilation during follow-up) and ICU enrollments (death or hospitalization ≥28 days).

### Statistical Analysis

Logistic regression was used to compare risk of disease progression outcomes among influenza type/subtypes. Crude and adjusted odds ratios (ORs) and 95% confidence intervals were derived using A(H1N1)pdm09 as the reference group. For FLU002 adjusted analyses, baseline covariates were age, gender, ethnicity, geographic region, symptom duration ≥6 days at enrollment, body mass index (BMI), smoking status, influenza vaccination in the season pre-enrollment (note that this study was not designed to assess vaccine efficacy), antiviral usage, and the presence of at least 1 of the following clinician-defined comorbidities: asthma/chronic obstructive pulmonary disease, immunosuppression, cardiovascular/liver/renal disease, or diabetes. Covariates significant in univariate analyses for FLU002 were retained for additional multivariable analysis in a parsimonious model. Covariates that remained significant in multivariable analysis were also considered for potential effect modification, ie, whether type/subtype differences varied by the significant predictor of outcome (for these, the interaction *P* value is cited).

FLU003 used the same covariates, along with the presence of at least 1 complication that initially defined enrollment eligibility. Crude and adjusted ORs were estimated for ward and ICU enrollments, separately and overall. For the latter, unadjusted and adjusted analyses were stratified according to ICU or general ward enrollment.

Sensitivity analyses were performed for both cohorts: 1 restricted to those enrolled post-April 2012 when all 3 viruses were co-circulating (to minimize confounding that might arise from combining analyses across seasons) and a second restricted to sites that enrolled at least 1 patient with each type/subtype. For FLU003, a sensitivity analysis was carried out for baseline and outcome variables in which viruses were compared for the time pre/post the October 2013 protocol amendment.

Following a significant (*P* < .05) omnibus test for virus differences in disease progression outcomes, pairwise comparisons of viruses were considered significant if the (2-sided) *P* value was less than .05. To reduce type 1 error risk for type/subtype comparisons for baseline characteristics, a *P* value for the omnibus test of .01 was used to assess the significance (*P* < .01) of pairwise comparisons. Analyses used SAS v. 9.4 (Statistical Analysis Software, Cary NC).

## RESULTS

**Figure 1.**
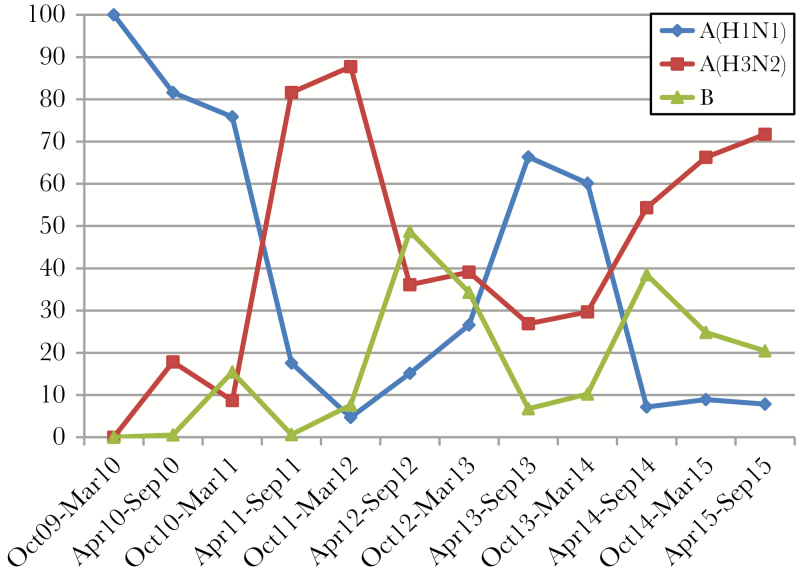
Prevalence of influenza virus type/influenza A virus subtypes, October 2009 through September 2015.

Over 6 years from October 2009 through September 2015, each year reflecting 1 northern and southern hemisphere winter, there were 5350 enrollments (3952 outpatients and 1398 inpatients) with laboratory-confirmed influenza: 1931 with A(H1N1)pdm09, 2389 with A(H3N2), and 1030 with influenza B. Initial seasons were dominated by A(H1N1)pdm09; 2011–2012 were dominated by A(H3N2), followed by substantial co-circulation of the 3 viruses (Figure 1).

In FLU002, 89 sites in 17 countries enrolled 9555 outpatients with ILI; 3952 (41.4%) were central laboratory-confirmed: 1290 (32.6%) with A(H1N1)pdm09, 1857 (47.0%) with A(H3N2), and 805 (20.4%) with B (Table 1, Supplementary Figure S1).

**Table 1.** Baseline Characteristics of FLU002 Outpatient and FLU003 Inpatient Cohort Enrollments and Influenza Virus Type/Influenza A Virus Subtype

	FLU002 (N = 3952)				FLU003 (N = 1398)			
	A(H1N1)pdm09	A(H3N2)	B		A(H1N1)pdm09	A(H3N2)	B	
Characteristics	n = 1290	n = 1857	n = 805	*P* Value^a^	n = 641	n = 532	n = 225	*P* Value^a^
Age, median (25th, 75th), y	35 (27, 47)	39 (30, 51)	42 (34, 53)	<.0001	49 (36, 61)	67 (49, 77)	59 (45, 71)	<.0001
18–34, %	50.9	39.3	28.7	<.0001	22.4	13.4	12.0	<.0001
35–49, %	30.7	34.0	40.3		30.3	12.2	19.1	
50–64, %	15.7	19.6	25.6		32.5	19.9	32.9	
65+, %	2.6	7.0	5.5		14.8	54.5	36.0	
Female, %	52.7	52.8	49.9	.36	53.2	51.9	52.4	.71
Race^b^				<.0001				.08
Asian, %	26.7	19.5	20.0		11.1	16.7	13.8	
Black, %	4.6	3.3	3.4		7.3	9.0	7.6	
White/other, %	68.7	77.1	76.6		81.6	74.2	78.7	
BMI, median (25th, 75th), kg/m^2^	24.6 (21.8, 28.1)	24.8 (22.0, 28.1)	25.1 (22.5, 28.8)	.03	26.2 (23.0, 30.6)	26.3 (22.7, 31.3)	26.0 (22.9, 30.5)	.62
Smoker, %	19.3	17.9	21.4	.12	29.4	16.3	19.1	<.0001
Pregnant, % of women aged ≤45 y	3.3	3.7	3.5	.94	24.8	23.5	38.5	.16
Vaccination in past 12 mo, %	13.9	19.1	16.0	.0005	26.2	49.6	36.4	<.0001
Antiviral therapy, %	1.9	1.0	0.7	.03	79.9	78.0	70.7	.03
Time since symptom onset, d				<.0001				<.0001
0–3 d	84.8	87.6	77.1		21.9	35.7	22.8	
4–5 d	10.5	9.2	15.0		24.3	26.1	24.2	
≥6 d	4.6	3.2	7.8		53.8	38.2	53.0	
Comorbidities								
Asthma/COPD, %	5.9	5.5	4.1	.19	30.3	38.9	28.4	.003
Diabetes, %	3.5	3.7	4.3	.58	12.8	23.9	16.4	<.0001
CVD/liver/renal disease, %	2.8	3.7	4.8	.05	21.5	38.9	34.7	<.0001
HIV/other immune dysfunction, %	10.1	11.5	16.0	.0002	14.0	11.3	16.9	.10
Any of above, %	19.5	21.2	25.7	.003	54.4	73.5	65.3	<.0001

Abbreviations: BMI, body mass index; COPD, chronic obstructive pulmonary disease; CVD, cardiovascular disease.

^a^Baseline characteristics for influenza types/subtypes were compared in each study using the 2 degrees of freedom chi-square test for categorical statistics and Kruskal-Wallis rank for continuous variables. For FLU 003, *P* values are with stratification by ward at enrollment.

^b^In FLU002, Asian sites enrolled 20% of patients, Australia 1%, Europe 29%, South America 42%, and North America (USA) 9%. In FLU003, Asian sites enrolled 11% of patients, Australia 13%, Europe 44%, South America 9%, and North America (USA) 23%.

In FLU003, 87 sites (50 also participated in FLU002) in 16 countries enrolled 2170 patients hospitalized with suspected influenza; 1398 (64.4%) patients had laboratory-confirmed influenza: 641 (45.8%) with A(H1N1)pdm09, 532 (38.1%) with A(H3N2), and 225 (16.1%) with B (Table 1, Supplementary Figure S2). One thousand one hundred eighty-six (84.8%) were enrolled in the general ward, and 212 (15.2%) were enrolled in the ICU.

### FLU002 Outpatient Cohort Baseline Characteristics

The median age of outpatients with A(H1N1)pdm09 was 35 years, compared with 39 years for those with A(H3N2) (*P* < .0001) and 42 years with B (*P* < .0001; *P* < .0001 also for A(H3N2) vs B). Most outpatients were enrolled within 3 days of ILI onset. This varied significantly among types/subtypes (*P* < .0001): More influenza B–infected outpatients were enrolled 4–5 days or ≥6 days from symptom onset than A(H1N1)pdm09 (*P* < .0001) or A(H3N2) (*P* < .0001) infections. Comorbidities were common, and more frequent in influenza B virus infection than A(H1N1)pdm09 (*P* = .0009) or A(H3N2) (*P* = .01). This difference was largely due to a greater percentage of immunosuppressed outpatients with influenza B (*P* = .0002) (Table 1). Age differences among type/subtypes persisted (*P* < .0001) after adjustment for other factors in Table 1; differences in the prevalence of comorbidities were reduced (*P* = .09 after adjustment).

### FLU003 Inpatient Cohort Baseline Characteristics

Tables 1 and Supplementary Table S1 summarize baseline characteristics for the 1398 hospitalized patients, which were similar for ward (1186) and ICU (212) enrollments. The median time from hospitalization to enrollment was 2 days for ward and 3 days for ICU enrollments. ICU patients included those admitted directly or general ward transfers (74/212, 34.9%).

Compared with FLU002 outpatients, FLU003 inpatients were older with more underlying comorbidities (Table 1). Among hospitalizations, those with A(H1N1)pdm09 were younger (49 years) than A(H3N2) (67 years; *P* < .0001) or influenza B (59 years; *P* < .0001). The time from symptom onset to hospitalization and enrollment varied significantly among types/subtypes (*P* < .0001), with more A(H3N2) patients enrolled within 6 days of symptoms onset than with A(H1N1)pdm09 (*P* < .0001) or B (*P* = .0003). Differences in age among type/subtype persisted (*P* < .0001) after adjustment for other factors in Table 1.

Percentages of hospitalized patients with comorbidities varied by virus (*P* < .0001). More patients with A(H3N2) (*P* < .0001) or B (*P* = .007) had comorbidities than those with A(H1N1)pdm09. This partially reflected age differences; after age adjustment, a significant difference remained in the percentage with comorbidities between A(H1N1)pdm09 and A(H3N2) (*P* = .0009), but not between A(H1N1)pdm09 and B (*P* = .36). With adjustment for all of the factors in Table 1, the difference among type/subtypes in the prevalence with comorbidities was no longer significant (*P* = .16).

Overall, and among hospital ward–enrolled patients, the percentage with complications varied among types/subtypes (*P* < .0001): More inpatients with A(H1N1)pdm09 had complications compared with those with A(H3N2) (*P* < .0001) or B (*P* < .0001) (Supplementary Table S2). In part, the greater percentage of A(H1N1)pdm09 inpatients with complications arose because the initial A(H1N1)pdm09-focused protocol required at least 1 complication to be present at enrollment. When this requirement was removed in October 2013, there were no significant differences in percentages of patients with complications by virus type/subtype. Suspected bacterial pneumonia was more common among all patients with A(H1N1)pdm09 virus infection (*P* = .001) (Supplementary Table S2).

Over 6 years, more A(H1N1)pdm09 patients were enrolled from the ICU (20.3%) than those with A(H3N2) (11.3%; *P* < .0001) or B (9.8%; *P* = .0004): This was evident both before and after the October 2013 protocol change, and was significant after this change (*P* = .0003) (Table 2). This trend was also evident for seasons from April 2012 when all viruses were co-circulating (18.4% for A(H1N1)pdm09, 10.6% for A(H3N2), and 9.6% for B; *P* = .004).

**Table 2.** Number and Percent of FLU003 Inpatients Enrolled From the ICU

	A(H1N1)pdm09		A(H3N2)		B		
	No.	%	No.	%	No.	%	*P* Value^a^
Enrolled before October 2013	93	21.1	30	15.6	12	17.1	.24
Enrolled after October 2013	37	18.4	30	8.8	10	6.5	.0003
Overall	130	20.3	60	11.3	22	9.8	<.0001

Abbreviation: ICU, intensive care unit.

^a^Chi-square test.

Other factors associated with ICU enrollment were female gender, symptom duration ≥6 days, the presence of complications, and continent of enrollment (there were more ICU admissions in North and South America). When these factors and calendar period of enrollment were taken into account, the adjusted ORs of ICU enrollment for A(H3N2) vs A(H1N1)pdm09, B vs A(H1N1)pdm09, and A(H3N2) vs B were 0.49 (95% confidence interval [CI], 0.33–0.72; *P* = .0003), 0.48 (95% CI, 0.29–0.81; *P* = .005), and 1.01 (95% CI, 0.58–1.75; *P* = .97), respectively.

### FLU002 Outpatient Cohort Day-14 Composite Outcomes

Overall, 3787 enrolled outpatients (95.8%) had known hospitalization status at day 14 postenrollment (Supplementary Figure S1). The percentage hospitalized or dying within 14 days varied significantly among types/subtypes (*P* < .0001): 3.4% for A(H1N1)pdm09, 0.7% for A(H3N2), and 2.2% for B (Table 3).

**Table 3.** Composite Outcome at 14 Days in the FLU002 Outpatient Cohort According to Influenza Virus Type/Influenza A Virus Subtype

	A(H1N1)pdm09 (n = 1235)		A(H3N2) (n = 1785)		B (n = 767)		
	No.	%	No.	%	No.	%	*P* Value^a^
Death	1	0.1	0	0.0	0	0.0	
Hospitalization	41	3.3	13	0.7	17	2.2	<.0001
Death or hospitalization	42	3.4	13	0.7	17	2.2	<.0001

^a^Two degrees of freedom chi-square test.

Compared with A(H1N1)pdm09, the unadjusted ORs of progressing to death or hospitalization were 0.21 for outpatients with A(H3N2) (95% CI, 0.11–0.39; *P* < .0001), and 0.64 (95% CI, 0.36–1.14; *P* = 0.13) for patients with influenza B. The OR for patients with A(H3N2) vs B was 0.32 (95% CI, 0.16–0.67; *P* = .002). The OR adjusted for age, comorbidities, and geographic region for A(H3N2) vs A(H1N1)pdm09 was 0.25 (95% CI, 0.13–0.48; *P* < .0001), for B vs A(H1N1)pdm09 it was 0.65 (95% CI, 0.35–1.17; *P* = .15), and for A(H3N2) vs B it was 0.39 (95% CI, 0.19–0.82; *P* = .01).

Of covariates considered in adjusted analyses, older age (*P* < .01), the presence of ≥1 comorbidities (*P* = .002), and continent of enrollment (Australia/Asia, North America, Europe, South America; *P* < .0001) were significantly related to death or hospitalization in both univariate and fully adjusted analyses. For each of the subgroups defined by these baseline factors, outpatients with A(H1N1)pdm09 were more likely to be hospitalized. Among older outpatients, the difference among type/subtypes was reduced, leading to a significant age interaction (*P* = .03) (Table S3).

A sensitivity analysis using enrollments post–April 2012, when there was substantial virus co-circulation, demonstrated similar findings to those enrolled over 6 years. The percentages of outpatients who were subsequently hospitalized were 2.0%, 0.9%, and 2.1% for A(H1N1)pdm09, A(H3N2), and B, respectively (*P* = .03). Compared with A(H1N1)pdm09, the unadjusted ORs of progressing to death or hospitalization were 0.42 for outpatients with A(H3N2) (95% CI, 0.19–0.92; *P* = .03) and 1.03 (95% CI, 0.49–2.14; *P* = .94) for patients with B. The OR for A(H3N2) vs B was 0.41 (95% CI, 0.19–0.88; *P* = .02).

A second sensitivity analysis was performed including sites that enrolled one or more outpatients with each type/subtype. In 3416 patients with 61 outcomes, the OR of A(H3N2) vs A(H1N1)pdm09 was 0.23 (95% CI, 0.12–0.45; *P* < .0001), for B vs A(H1N1)pdm09 it was 0.68 (95% CI, 0.37–1.25; *P* = .21), and for A(H3N2) vs B it was 0.34 (95% CI, 0.16–0.72; *P* = .004).

### FLU003 Inpatient Cohort Day-60 Composite Outcomes

Day 60 disease progression outcomes were known for 1291 (92.3%) inpatients (Supplementary Figure S2): The percentage with a composite outcome did not vary by virus (Table 4). This was observed overall (*P* = .32) and for ICU (*P* = .80) or ward (*P* = .39) enrollments. With considerations of the components of the composite outcome, there was no difference in the number of patients transferred to the ICU from the ward (*P* = .24) while there was a trend toward more patients with A(H1N1)pdm09 having an extended (≥28 days) hospitalization (*P* = .07); the percentage dying within 60 days was 7.1% for A(H1N1)pdm09, 5.0% for A(H3N2), and 7.3% for B (*P* = .30). Of the 82 deaths, 64 (78.0%) occurred during the enrollment hospitalization. Mortality was higher in ICU (24.3%) than general ward enrollments (3.0%; *P* < .001).

**Table 4.** Composite Outcome at 60 Days in the FLU003 Inpatient Cohort According to Influenza Virus Type/Influenza A Virus Subtype

	A(H1N1)pdm09		A(H3N2)		B		
	No.	%·	No.	%	No.	%	*P* Value^a^
Enrollment From General Ward	n = 467		n = 441		n = 185		
Composite outcome^b^	54	11.6	39	8.8	18	9.7	.39
Death	15	3.2	11	2.5	7	3.8	.65
Extended hospitalization^c^	23	4.5	17	3.6	5	2.5	.42
Transfer to ICU^d^	30	5.9	17	3.6	11	5.4	.24
Enrollment from ICU	n = 120		n = 58		n = 20		
Composite outcome^b^	58	48.3	25	43.1	9	45.0	.80
Death	27	22.1	14	23.7	8	38.1	.29
Extended hospitalization^c^	37	29.·6	11	18.6	3	14.3	.14
Overall	n = 587		n = 499		n = 205		
Composite outcome^b^	112	19.1	64	12.8	27	13.2	.32
Death	42	7.1	25	5.0	15	7.3	.30
Extended hospitalization^c^	60	9.5	28	5.3	8	3.6	.07

Abbreviation: ICU, intensive care unit.

^a^Two degrees of freedom chi-square test. Overall is stratified by ward at enrollment.

^b^Death, extended hospitalization, or progression to ICU or mechanical ventilation.

^c^Hospitalized for ≥28 days after enrollment.

^d^If enrolled from the general ward, progression to ICU or mechanical ventilation.

Compared with A(H1N1)pdm09 virus-infected inpatients, the adjusted ORs of death, hospitalization ≥28 days, ICU admission, or use of mechanical ventilation were 0.87 for A(H3N2) (95% CI, 0.52–1.44; *P* = .59) and 1.00 (95% CI, 0.57–1.75; *P* = 1.00) for influenza B; the OR for A(H3N2) vs B was 0.87 (95% CI, 0.48–1.56; *P* = .64) (Table 5). In analyses based on seasons post–April 2012 when all viruses were co-circulating, the adjusted ORs of death, hospitalization ≥28 days, ICU admission, or use of mechanical ventilation were 1.08 for A(H3N2) vs A(H1N1) (95% CI, 0.57–2.06; *P* = .82), 1.28 for B vs A(H1N1) (95% CI, 0.65–2.55; *P* = .47), and 0.84 (95% CI, 0.46–1.54; *P* = .57) for A(H3N2) vs B. Of covariates considered in the adjusted analysis, in univariate analyses (stratified by enrollment location only), age (*P* < .0001), lower BMI (*P* = .003), and continent of enrollment (Europe and South America; *P* = .0009) were associated with an increased risk of disease progression over the 60-day follow-up. Virus differences in the composite outcome and in death were consistent among subgroups defined by age, continent of enrollment, presence of comorbidities, and complications (Tables S4 and S5).

**Table 5.** Unadjusted and Adjusted Odds Ratios Disease Progression in the FLU003 Inpatient Cohort by Influenza Virus Type/Influenza A Virus Subtype

	A(H3N2) vs A(H1N1)pdm09			B vs A(H1N1)pdm09			A(H3N2) vs B		
Enrolled From General Ward	OR	95% CI	*P* Value	OR	95% CI	*P* Value	OR	95% CI	*P* Value
Unadjusted^a^	0.74	0.48–1.15	.18	0.82	0.47–1.45	.50	0.90	0.50–1.62	.73
Adjusted^b^	0.79	0.44–1.43	.43	0.95	0.49–1.84	.89	0.83	0.43–1.59	.57
Enrolled from ICU									
Unadjusted^a^	0.81	0.43–1.52	.51	0.87	0.34–2.26	.78	0.93	0.33–2.57	.88
Adjusted^b^	0.11	0.38–3.25	.85	1.06	0.30–3.83	.92	1.05	0.25–4.45	.95
Total									
Unadjusted^a^	0.76	0.53–1.09	.14	0.84	0.52–1.36	.48	0.91	0.55–1.51	.71
Adjusted^b^	0.87	0.52–1.44	.59	1.00	0.57–1.75	1.00	0.87	0.48–1.56	.64

Abbreviations: CI, confidence interval; ICU, intensive care unit; OR, odds ratio.

^a^Overall is stratified by hospital unit at enrollment.

^b^Stratified by hospital unit at enrollment and adjusted for region, age, gender, ethnicity, body mass index, smoking, duration of symptoms, ≥1 comorbidities, ≥1 severe complications, influenza vaccine in past year, and use of antivirals on or before enrollment date.

## DISCUSSION

In these 2 large international cohort studies of adults with laboratory-confirmed influenza, outpatients with A(H1N1)pdm09 were more likely to be hospitalized within 14 days than those with A(H3N2) or influenza B. Although hospitalized adults with A(H1N1)pdm09 were more likely to have severe influenza as measured by the percentage of ICU enrollments, differences in disease progression or mortality at 60-day follow-up were not detected. Notably, both outpatients and hospitalized patients with A(H3N2) or B had more comorbidities than those with A(H1N1)pdm09, and patients with A(H1N1)pdm09 were younger.

Among outpatients, the 14-day hospitalization risk was lowest for those with influenza A(H3N2). The percentage of outpatients with A(H3N2) subsequently hospitalized was 0.7%, compared with 3.4% and 2.2% for A(H1N1)pdm09 and B, respectively. The lower A(H3N2) hospitalization risk persisted after adjustment for age and other covariates, when analyses were done over influenza seasons with substantial virus co-circulation and for sites that enrolled at least 1 patient of each type/subtype. The finding was also consistent for subgroups defined by age, continent of enrollment, and presence of comorbidities. Our results differ from studies that examined hospitalization within 30 days among enrollments in vaccine effectiveness studies in Wisconsin [5,6]. The differences may relate to variations in sample size and statistical power, the study time periods, inclusion of children, outcome definitions, or unmeasured confounders. In Belongia et al. [6], 6/150 (4.0%) adults with A(H1N1)pdm09 were hospitalized within 30 days of onset (contrasting with 14-day follow-up in FLU002) vs 17/377 (4.5%) for A(H3N2). However, outpatients with A(H1N1)pdm09 were enrolled in 2009, and those with A(H3N2) were enrolled in 2007–2008 (influenza B patients were not analyzed) [6], whereas we enrolled adults continuously from 2009 through 2015.

In FLU003, similar to US Centers for Disease Control and Prevention (CDC) studies [2, 3], we found that inpatients with A(H1N1)pdm09 were more likely to be admitted to the ICU. Although more A(H1N1)pdm09 inpatients were enrolled from the ICU (and the percentage transferring to the ICU from the general ward was higher), this difference was not significant. If ICU enrollment and transfer from the ward are considered together, A(H1N1)pdm09 virus infection appeared to be associated with more severe disease.

The observation of increased severity of hospitalization with A(H1N1)pdm09 virus infection was not supported by the 60-day follow-up. Disease progression did not vary among types/subtypes irrespective of general ward or ICU enrollment. Furthermore, in neither the CDC studies [2, 6] nor FLU003 was there evidence of mortality differences among types/subtypes. In Chaves et al. [2], the percentages of patients dying before hospital discharge were 3.7%, 3.9%, and 4.1%, for A(H1N1)pdm09, A(H3N2), and influenza B, respectively. In FLU003 inpatients, the corresponding percentages for death within 60 days were 7.1% (A(H1N1)pdm09), 5.0% (A(H3N2)), and 7.3% (B). Two other studies have reported mortality by virus type/subtype. Loubet et al. [4] reported that the percentage of patients dying during hospitalization did not vary by type/subtype (A(H1N1)pdm09 2%, A(H3N2) 4%, influenza B 4%), but the total number of deaths (n = 20) was much smaller than in FLU003 (n = 82) or Chaves et al. (n = 109) [2]. Similarly, in Hong Kong mortality was compared for adults hospitalized with influenza A vs B in 2007–2008 and found not to differ: 21/414 (5%) with A(H3N2) vs 13/215 (6%) with influenza B [9]. It is uncertain why outpatients with A(H1N1)pdm09 were more likely to be hospitalized, but hospitalized A(H1N1)pdm09-infected patients did not have worse 60-day outcomes (despite being more likely to be enrolled from the ICU). One explanation may be that younger adults hospitalized with A(H1N1)pdm09 virus infection, although less likely to have comorbidities, were more likely to be admitted to the ICU if complications were present.

Strengths of our study include concurrent, prospective enrollment of both outpatients and inpatients from multiple countries in both hemispheres over 6 years. A defined follow-up period for assessing outcomes was used with a high proportion of follow-up evaluation, and common protocols were used for sample collection and laboratory testing. Limitations include adults-only enrollment and a relatively small number of disease progression outcomes in the outpatient cohort, thereby limiting power, an issue identified for many studies [10].

In summary, the findings from these global follow-up studies illustrate the benefit of planned prospective observational studies to complement influenza surveillance approaches. Such studies are critical for assessing predictors of disease progression including the changing severity among types/subtypes over time.

## Supplementary Data

Supplementary materials are available at *Open Forum Infectious Diseases* online. Consisting of data provided by the authors to benefit the reader, the posted materials are not copyedited and are the sole responsibility of the authors, so questions or comments should be addressed to the corresponding author.

## Supplementary Material

ofx212_suppl_supplementary_table_s1

ofx212_suppl_supplementary_table_s2

ofx212_suppl_supplementary_figure_s1

ofx212_suppl_supplementary_figure_s2
