# Supplementary material for: Comparison of the Outcomes of Individuals With Medically Attended Influenza A and B Virus Infections Enrolled in 2 International Cohort Studies Over a 6-Year Period: 2009–2015
Source: Open Forum Infect Dis. 2017 Oct 7;4(4):ofx212. doi: 10.1093/ofid/ofx212 (PMC5740982; doi:10.1093/ofid/ofx212)
Supplement: ofx212_suppl_supplementary_table_s1 [file ofx212_suppl_supplementary_table_s1.docx]

**Table S1. Baseline Characteristics of FLU003 Inpatient Cohort Enrollments by Ward of Admission and Influenza Virus Type/Influenza A Virus Subtype**

|  |  | **General Ward (N=1186)** | | | |  | **ICU (N=212)** | | | |
| --- | --- | --- | --- | --- | --- | --- | --- | --- | --- | --- |
|  |  | **A(H1N1)pdm09** | **A(H3N2)** | **B** |  |  | **A(H1N1)pdm09** | **A(H3N2)** | **B** |  |
| **Characteristics** |  | **N=511** | **N=472** | **N=203** | **P-value**^a^ |  | **N=130** | **N=60** | **N=22** | **P-value**^a^ |
| Age – median  (25^th^, 75^th^) years |  | 49  (36, 61) | 67 (49, 77) | 60  (46, 71) | <0·0001 |  | 49  (37, 59) | 66  (50, 75) | 51  (36, 69) | <0·0001 |
| 18-34 (%)  35-49 (%)  50-64 (%)  65+ (%) |  | 22·5  29·9  31·9  15·7 | 13·3  12·3  19·9  34·1 | 10·8  18·2  35·0  36·0 | <0·0001 |  | 22·3 31·5 34·6 11·5 | 13·3  11·7  20·0  55·0 | 22·7  27·3  13·6  36·4 | <0·0001 |
| Female (%) |  | 55·6 | 52·5 | 54·7 | 0·63 |  | 43·8 | 46·7 | 31·8 | 0·48 |
| Race |  |  |  |  | 0·01 |  |  |  |  | 0·11 |
| Asian (%) |  | 11·0 | 18·4 | 15·3 |  |  | 11·5 | 3·3 | 0·0 |  |
| Black (%) |  | 7·8 | 9·1 | 6·9 |  |  | 5·4 | 8·3 | 13·6 |  |
| White/other (%) |  | 81·2 | 72·5 | 77·8 |  |  | 83·1 | 88·3 | 86·4 |  |
| BMI – median (25^th^, 75^th^) kg/m^2^ |  | 25·8  (22·7, 30·3) | 26·2  (22·5, 31·3) | 26·0  (22·7, 30·4) | 0·62 |  | 27·3  (24·2, 31·7) | 26·7  (23·9, 31·3) | 26·9  (24·5, 31·3) | 0·98 |
| Smoker (%) |  | 30·0 | 15·4 | 19·1 | <0·0001 |  | 26·8 | 23·6 | 19·0 | 0·72 |
| Pregnant (% of women ≤45 years) |  | 20·0 | 22·2 | 36·0 | 0·21 |  | 50·0 | 40·0 | 100·0 | 0·56 |
| Vaccination in past 12 months (%) |  | 27·7 | 50·0 | 36·9 | <0·001 |  | 19·0 | 46·3 | 31·2 | 0·001 |
| Antiviral therapy (%) |  | 78·7 | 77·3 | 69·5 | 0·03 |  | 84·6 | 83·3 | 81·8 | 0·94 |
| Time since symptoms onset (days) |  |  |  |  | <0·0001 |  |  |  |  | 0·0003 |
| 0-3 days |  | 24·6 | 37·2 | 22·3 |  |  | 10·7 | 23·2 | 27·3 |  |
| 4-5 days |  | 27·0 | 25·1 | 25·4 |  |  | 13·1 | 33·9 | 13·6 |  |
| ≥6 days |  | 48·3 | 37·7 | 52·3 |  |  | 76·2 | 42·9 | 59·1 |  |
| Comorbidities |  |  |  |  |  |  |  |  |  |  |
| Asthma/COPD (%) |  | 33·3 | 38·8 | 28·6 | 0·03 |  | 18·5 | 40·0 | 27·3 | 0·007 |
| Diabetes (%) |  | 11·7 | 24·1 | 15·8 | <0·0001 |  | 16·9 | 21·7 | 22·7 | 0·66 |
| CVD/liver/renal disease (%) |  | 21·5 | 37·1 | 35·0 | <0·0001 |  | 21·5 | 53·3 | 31·8 | <0·0001 |
| HIV/other immune dysfunction (%) |  | 13·7 | 10·0 | 17·2 | 0·03 |  | 15·4 | 21·7 | 13·6 | 0·51 |
| Any of above (%) |  | 55·4 | 72·2 | 66·0 | <0·0001 |  | 50·8 | 83·3 | 59·1 | 0.0001 |
| ^a^2df Chi-square test for categorical and Kruskal-Wallis for continuous variables. | | | | | | | | | | |
