# Supplementary material for: Comparison of the Outcomes of Individuals With Medically Attended Influenza A and B Virus Infections Enrolled in 2 International Cohort Studies Over a 6-Year Period: 2009–2015
Source: Open Forum Infect Dis. 2017 Oct 7;4(4):ofx212. doi: 10.1093/ofid/ofx212 (PMC5740982; doi:10.1093/ofid/ofx212)
Supplement: ofx212_suppl_supplementary_table_s2 [file ofx212_suppl_supplementary_table_s2.docx]

**Table S2. Complications Experienced by Enrollments in the FLU003 Inpatient Cohort**

|  |  | **A (H1N1)pdm09** | |  | **A(H3N2)** | |  | **B** | |  | **P-value**^a^ |
| --- | --- | --- | --- | --- | --- | --- | --- | --- | --- | --- | --- |
| **Enrolled from General Ward** |  | **No.** | **%** |  | **No.** | **%** |  | **No.** | **%** |  |  |
| ***Severe or complicated influenza***^b^ | | | | | | | | | | | |
| Respiratory distress requiring O_2_ |  | 333 | 65·2 |  | 190 | 40·3 |  | 89 | 43·6 |  | <0·0001 |
| Exacerbation of pre-existing medical condition |  | 185 | 36·2 |  | 162 | 34·3 |  | 71 | 35·0 |  | 0·82 |
| Other severe complication |  | 82 | 16·0 |  | 80 | 16·9 |  | 33 | 16·3 |  | 0·93 |
| Any above complications |  | 453 | 88·6 |  | 331 | 70·1 |  | 139 | 68·5 |  | <0·0001 |
| *Other complications* |  |  |  |  |  |  |  |  |  |  |  |
| Dehydration requiring IV hydration |  | 170 | 33·3 |  | 149 | 31·6 |  | 66 | 32·5 |  | 0·85 |
| Bacterial pneumonia |  | 122 | 23·9 |  | 65 | 13·8 |  | 28 | 13·8 |  | <0·0001 |
| **Enrolled from ICU** |  |  |  |  |  |  |  |  |  |  |  |
| ***Severe or complicated influenza***^b^ | | | | | | | | | | | |
| Respiratory distress requiring O_2_ |  | 111 | 85·4 |  | 41 | 68·3 |  | 22 | 100·0 |  | 0·001 |
| Exacerbation of pre-existing medical condition |  | 26 | 20·0 |  | 28 | 46·7 |  | 5 | 22·7 |  | 0·0006 |
| Other severe complication |  | 66 | 50·8 |  | 25 | 41·7 |  | 11 | 50·0 |  | 0·50 |
| Any above complications |  | 123 | 94·6 |  | 54 | 90·0 |  | 22 | 100·0 |  | 0·21 |
| *Other complications* |  |  |  |  |  |  |  |  |  |  |  |
| Dehydration requiring IV hydration |  | 52 | 40·0 |  | 22 | 36·7 |  | 5 | 22·7 |  | 0·30 |
| Bacterial pneumonia |  | 49 | 37·7 |  | 28 | 46·7 |  | 7 | 31·8 |  | 0·37 |
| **Total** |  |  |  |  |  |  |  |  |  |  |  |
| ***Severe or complicated influenza***^b^ |  |  |  |  |  |  |  |  |  |  |  |
| Respiratory distress requiring O_2_ |  | 444 | 69·3 |  | 231 | 43·4 |  | 111 | 49·3 |  | <0·0001 |
| Exacerbation of pre-existing medical condition |  | 211 | 32·9 |  | 190 | 35·7 |  | 76 | 33·3 |  | 0·72 |
| Other severe complication |  | 148 | 23·1 |  | 105 | 19·7 |  | 44 | 19·6 |  | 0·98 |
| Any above complications |  | 576 | 89·9 |  | 385 | 72·4 |  | 161 | 71·6 |  | <0·0001 |
| *Other complications* |  |  |  |  |  |  |  |  |  |  |  |
| Dehydration requiring IV hydration |  | 222 | 34·6 |  | 171 | 32·1 |  | 71 | 31·6 |  | 0·67 |
| Bacterial pneumonia |  | 171 | 26·7 |  | 93 | 17·5 |  | 35 | 15·6 |  | 0·001 |
| ^a^Chi-square test. Total is stratified by ward.  ^b^Eligibility criteria through August 2013. | | | | | | | | | | | |

**Table S3. Hospitalization or Death for FLU002 Outpatients According to Influenza Virus Type/Influenza A Virus Subtype for Baseline Defined Subgroups**

|  |  | **A (H1N1)pdm09** | |  | **A(H3N2)** | |  | **B** | |  | **Interaction P-value** |
| --- | --- | --- | --- | --- | --- | --- | --- | --- | --- | --- | --- |
| **Subgroup** |  | **No.** | **%** |  | **No.** | **%** |  | **No.** | **%** |  |  |
| Age | | | | | | | | | | | |
| 18-34 |  | 20 | 3·2 |  | 5 | 0·7 |  | 2 | 0·9 |  | 0·03 |
| 35-49 |  | 12 | 3·2 |  | 1 | 0·2 |  | 5 | 1·6 |  |  |
| 50+ |  | 10 | 4·4 |  | 7 | 1·5 |  | 10 | 4·1 |  |  |
| Continent of enrollment | | | | | | | | | | | |
| North America |  | 11 | 6·6 |  | 2 | 1·8 |  | 1 | 2·8 |  | 0·51 |
| Europe |  | 20 | 4·3 |  | 5 | 1·3 |  | 12 | 5·0 |  |  |
| South America |  | 4 | 1·3 |  | 1 | 0·1 |  | 2 | 0·6 |  |  |
| Australia/Asia |  | 7 | 2·3 |  | 5 | 1·4 |  | 2 | 1·3 |  |  |
| Underlying chronic condition (comorbidity) | | | | | |  |  |  |  |  |  |
| Yes |  | 14 | 5·8 |  | 9 | 2·4 |  | 6 | 3·0 |  | 0·08 |
| No |  | 28 | 2·8 |  | 4 | 0·3 |  | 11 | 1·9 |  |  |

**Table S4. Progression of Disease for FLU003 Inpatients According to Influenza Virus Type/Influenza A Virus Subtype for Baseline Defined Subgroups**

|  |  | | **A(H1N1)pdm09** | | |  | **A(H3N2)** | |  | **B** | |  | **Interaction P-value**^a^ |
| --- | --- | --- | --- | --- | --- | --- | --- | --- | --- | --- | --- | --- | --- |
| **Subgroup** | |  | **No.** | **%** |  | | **No.** | **%** |  | **No.** | **%** |  |  |
| Age | | | | | | | | | | | | | |
| 18-34 | |  | 17 | 12·8 |  | | 5 | 7·5 |  | 5 | 19·2 |  | 0·83 |
| 35-49 | |  | 35 | 19·9 |  | | 1 | 1·8 |  | 5 | 12·8 |  |  |
| 50+ | |  | 60 | 21·6 |  | | 58 | 15·4 |  | 17 | 12·1 |  |  |
| Continent of Enrollment | | | | | | | | | | | | | |
| North America | |  | 13 | 12·6 |  | | 17 | 10·8 |  | 2 | 5·4 |  | 0·34 |
| Europe | |  | 74 | 21·1 |  | | 22 | 16·4 |  | 13 | 16·0 |  |  |
| South America | |  | 6 | 22·2 |  | | 14 | 19·2 |  | 7 | 36·8 |  |  |
| Australia/Asia | |  | 19 | 17·9 |  | | 11 | 8·2 |  | 5 | 7·4 |  |  |
| Underlying Chronic Condition (comorbidity) | | | | | | | |  |  |  |  |  |  |
| Yes | |  | 58 | 18·3 |  | | 55 | 15·0 |  | 17 | 12·9 |  | 0·19 |
| No | |  | 54 | 20·0 |  | | 9 | 6·8 |  | 10 | 13·7 |  |  |
| Serious Complications^a^ | | | | | | | | | | | | | |
| Yes | |  | 110 | 20·7 |  | | 51 | 14·3 |  | 20 | 14·2 |  | 0·08 |
| No | |  | 2 | 3·6 |  | | 13 | 9·2 |  | 7 | 10·9 |  |  |
|  | |  |  |  |  | |  |  |  |  |  |  |  |
| ^a^Stratified by ward of enrollment.  ^b^Complications defining eligibility. | | | | | | | | | | | | | |

**Table S5. Death for FLU003 Inpatients According to Influenza Virus Type/Influenza A Virus Subtype for Baseline Defined Subgroups**

|  |  | **A(H1N1)pdm09** | |  | **A(H3N2)** | |  | **B** | |  | **Interaction P-value**^a^ |
| --- | --- | --- | --- | --- | --- | --- | --- | --- | --- | --- | --- |
| **Subgroup** |  | **No.** | **%** |  | **No.** | **%** |  | **No.** | **%** |  |  |
| Age | | | | | | | | | | | |
| 18-34 |  | 6 | 4·5 |  | 0 | 0·0 |  | 1 | 3·8 |  | 0·05 |
| 35-49 |  | 12 | 6·9 |  | 0 | 0·0 |  | 3 | 7·7 |  |  |
| 50+ |  | 24 | 8·6 |  | 25 | 6·6 |  | 11 | 7·8 |  |  |
| Continent of Enrollment | | | | | | | | | | | |
| North America |  | 3 | 2·9 |  | 6 | 3·8 |  | 1 | 2·7 |  | 0·85 |
| Europe |  | 30 | 8·6 |  | 6 | 4·5 |  | 8 | 9·9 |  |  |
| South America |  | 3 | 10·7 |  | 10 | 13·5 |  | 4 | 20·0 |  |  |
| Australia/Asia |  | 6 | 5·6 |  | 3 | 2·2 |  | 2 | 2·9 |  |  |
| Underlying Chronic Condition (comorbidity) | | | | | |  |  |  |  |  |  |
| Yes |  | 25 | 7·8 |  | 24 | 6·5 |  | 9 | 6·8 |  | 0·20 |
| No |  | 17 | 6·3 |  | 1 | 0·8 |  | 6 | 8·1 |  |  |
| Serious Complications^b^ | | | | | | | | | | | |
| Yes |  | 42 | 7·9 |  | 19 | 5·3 |  | 11 | 7·7 |  | 0·95 |
| No |  | 0 | 0·0 |  | 6 | 4·2 |  | 4 | 6·3 |  |  |
|  |  |  |  |  |  |  |  |  |  |  |  |
| ^a^Stratified by ward of enrollment.  ^b^Complications defining eligibility. | | | | | | | | | | | |
