# Supplementary material for: Comparison of the Outcomes of Individuals With Medically Attended Influenza A and B Virus Infections Enrolled in 2 International Cohort Studies Over a 6-Year Period: 2009–2015
Source: Open Forum Infect Dis. 2017 Oct 7;4(4):ofx212. doi: 10.1093/ofid/ofx212 (PMC5740982; doi:10.1093/ofid/ofx212)
Supplement: ofx212_suppl_supplementary_figure_s1 [file ofx212_suppl_supplementary_figure_s1.docx]

**Figure S1. FLU002 Outpatient Cohort Flow Diagram**

Patients enrolled from October 2009 through September 2015
N=9555

5603 (58·6%) excluded
- 2 (<0·1%) withdrew consent - 5527 (57·8%) RT-PCR negative - 3 (<0·1%) influenza virus co-infection
- 71 (0·7%) influenza status unknown*

Disease Progression at 14 days
N=72 (1· 8%)

Patients with RT-PCR confirmed influenza A(H1N1)pdm09, A(H3N2) or B
N=3952

No Disease Progression at 14 days
N=3715 (94·0%)

Disease Progression status unknown**
 N=165 (4·2%)
A(H1N1)pdm09 N=55 (4·3%) A(H3N2) N=72 (3·9%) influenza B N=38 (4·7%)

* Lost specimen, sample collection problem, or uninterpretable result.

** Disease progression status was considered unknown if hospitalization status or vital status data on day 14 was unavailable.

The FLU002 protocol is available at <http://insight.ccbr.umn.edu/official_documents/FLU002/protocol_documents/FLU002_Protocol.pdf>.
