# Supplementary material for: Comparison of the Outcomes of Individuals With Medically Attended Influenza A and B Virus Infections Enrolled in 2 International Cohort Studies Over a 6-Year Period: 2009–2015
Source: Open Forum Infect Dis. 2017 Oct 7;4(4):ofx212. doi: 10.1093/ofid/ofx212 (PMC5740982; doi:10.1093/ofid/ofx212)
Supplement: ofx212_suppl_supplementary_figure_s2 [file ofx212_suppl_supplementary_figure_s2.docx]

**Figure S2. FLU003 Inpatient Cohort Flow Diagram**

Patients enrolled from October 2009 through September 2015
N=2170

772 (35·6%) excluded
 - 15 (0·7%) withdrew consent
 - 639 (29·4%) RT-PCR negative for influenza A and B - 93 (4·3%) RT-PCR positive for influenza A, subtype undetermined
 - 25 (1·2%) influenza status unknown*

Disease Progression at 60 days
N=203 (14·5%)

Patients with RT-PCR confirmed influenza A(H1N1)pdm09, A(H3N2) or B
N=1398

No Disease Progression at 60 days
N=1088 (77·8%)

Disease Progression status unknown**
N=107 (7·7%)
A(H1N1)pdm09 N=54 (8·4%) A(H3N2) N=33 (6·2%)
influenza B N=20 (8·9%)

* Lost specimen, sample collection problem, or uninterpretable result.

** Disease progression status was considered unknown if death, extended hospitalization (≥28 days), or progression to ICU or mechanical ventilation data on day 60 was unavailable.

The FLU003 protocol is available at http://insight.ccbr.umn.edu/official_documents/FLU003/protocol_documents/FLU003_Protocol.pdf.
